# Optimal treatment strategy with nilotinib for patients with newly diagnosed chronic‐phase chronic myeloid leukemia based on early achievement of deep molecular response (MR^4.5^): The phase 2, multicenter N‐Road study

**DOI:** 10.1002/cam4.3034

**Published:** 2020-04-06

**Authors:** Kaichi Nishiwaki, Kei‐ji Sugimoto, Shigehisa Tamaki, Junichi Hisatake, Hisayuki Yokoyama, Tadahiko Igarashi, Atsushi Shinagawa, Takeaki Sugawara, Satoru Hara, Kazuhisa Fujikawa, Seiichi Shimizu, Toshiaki Yujiri, Arinobu Tojo, Hisashi Wakita

**Affiliations:** ^1^ Division of Oncology and Hematology Jikei University Kashiwa Hospital Kashiwa Japan; ^2^ Division of Hematology Juntendo University Urayasu Hospital Chiba Japan; ^3^ Department of Hematology/Infectious Disease Ise Red Cross Hospital Ise Japan; ^4^ Department of Hematology Omori Red Cross Hospital Tokyo Japan; ^5^ Department of Hematology National Hospital Organization Sendai Medical Center Sendai Japan; ^6^ Division of Hematology and Oncology Gunma Cancer Center Ohta Japan; ^7^ Department of Internal Medicine Hitachi General Hospital Ibaraki Japan; ^8^ Division of Hematology‐Oncology Chiba Cancer Center Chiba Japan; ^9^ Department of Hematology Chiba Rosai Hospital Chiba Japan; ^10^ Department of Hematology Chibaken Saiseikai Narashino Hospital Narashino Japan; ^11^ Department of Hematology Tsuchiura Kyodo General Hospital Tsuchiura Japan; ^12^ Third Department of Internal Medicine Yamaguchi University Yamaguchi Japan; ^13^ Division of Molecular Therapy Institute of Medical Science Tokyo University Tokyo Japan; ^14^ Division of Hematology and Oncology Japanese Red Cross Society Narita Red Cross Hospital Narita Japan

**Keywords:** chronic myeloid leukemia, early deep molecular response, nilotinib

## Abstract

For patients who have chronic myeloid leukemia (CML), one of the primary treatment options is administration of nilotinib 300 mg twice daily (BID). In previous studies which compared outcomes associated with nilotinib or imatinib treatment, nilotinib achieved a higher rate of deep molecular response (MR). We conducted a phase II, open‐label, multicenter study to investigate an intrapatient nilotinib dose‐escalation strategy for patients with newly diagnosed chronic‐phase (CP) CML based on early MR^4.5^ achievement. The primary study endpoint was achievement of MR^4.5^ by 24 months following the initiation of nilotinib 300 mg BID. Fifty‐three patients were enrolled, 51 received nilotinib, and 37 completed the treatment. An increase in the nilotinib dose (to 400 mg BID) was allowed when patients satisfied our criteria for no optimal response at any time point. The median (range) dose intensity was 600 (207‐736) mg/day. Of 46 evaluable patients, 18 achieved an optimal response and 28 did not. Of the latter, nine patients underwent dose escalation to 400 mg BID, and none achieved MR^4.5^. The remaining 19 patients could not undergo dose escalation, 12 (63%) because of adverse events (AEs), and 7 (37%) for non‐AE related reasons. Four of these patients achieved MR^4.5^. The MR^4.5^ rate by 24 months was 45.7%. The progression‐free, overall and event‐free survival were each 97.6%. No new safety concerns were observed. Our findings support the use of continuous nilotinib at a dose of 300 mg BID for newly diagnosed patients with CML‐CP.

## Graphical abstract

The N‐Road study aimed to determine the optimal treatment strategy for nilotinib use in patients with newly diagnosed CML‐CP based on early achievement of deep molecular response. Most patients received nilotinib at a dose of 300 mg BID and were able to achieve an early deep molecular response. Together with the finding that none of the patients whose dose was escalated to 400 mg BID achieved MR^4.5^, we consider that continuous nilotinib at a dose of 300 mg BID may be the optimal treatment strategy for newly diagnosed patients with CML‐CP.

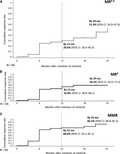

## 1 INTRODUCTION

The myeloproliferative disorder chronic myeloid leukemia (CML) is characterized by the acquisition of the Philadelphia chromosome (t[9;22][q34.1;q11.2]) in multipotent hematopoietic stem cells.^1^ Approximately 15% to 20% of leukemia cases are CML, that is, one out of 100,000 leukemia cases. The *BCR‐ABL1* fusion gene in the Philadelphia chromosome encodes and produces the BCR‐ABL tyrosine kinase; in CML, this tyrosine kinase is constantly activated. This leads to leukemia cell proliferation and disease progression.^2^, ^3^, ^4^ Without any treatment, CML in the chronic‐phase (CP) progresses to the accelerated phase (AP) or blast phase (BP). Both AP and BP are usually associated with a lethal outcome within 5 years. Initial treatment of CML‐CP consists of the use of one of the following three tyrosine kinase inhibitors (TKIs) as frontline therapy: imatinib, dasatinib, or nilotinib.^5^ The first approved TKI for the treatment of newly diagnosed CML‐CP was imatinib, based on clinical trial results in which imatinib and interferon‐α plus cytarabine were compared; the study data showed that imatinib had superior efficacy without any significant changes in the safety profile.^6^ Subsequent to the approval of imatinib,^6^, ^7^, ^8^, ^9^ the second‐generation TKIs dasatinib and nilotinib^10^, ^11^ were also approved for therapeutic use. Compared with imatinib, nilotinib has been shown to have a higher binding affinity and greater selectivity for the ABL kinase.^12^ In terms of its inhibitory activity, in imatinib‐sensitive CML cell lines, nilotinib is 20‐50 times more inhibitory than imatinib, and in imatinib‐resistant cell lines, nilotinib is 3‐7 times more inhibitory.^13^, ^14^, ^15^, ^16^, ^17^ For patients with newly diagnosed CML‐CP, the dose of nilotinib recommended in the package insert is 300 mg twice daily (BID).^18^

The ENESTnd study compared the efficacy and safety of nilotinib at two doses (300 mg BID and 400 mg BID) and of imatinib 400 mg QD, in newly diagnosed CML‐CP patients.^19^ At 2 years, frontline nilotinib at a dose of 300 mg BID resulted in a higher rate of deep molecular response (MR) compared with imatinib (MR 4.5 log reduction [MR^4.5^] by 2 years: nilotinib, 26% vs imatinib, 10%; *P* < .0001).^19^ By 5 years, more than half of all patients in each nilotinib arm (300 mg BID, 54%; 400 mg BID, 52%) achieved MR^4.5^ compared with 31% of patients in the imatinib arm.^19^ A phase II study in untreated CML patients evaluated 73 early CP individuals to whom nilotinib was administered at a dose of 400 mg BID. The report from that study indicated that patients were able to achieve a complete cytogenetic response (CCyR) rate of 96% by 1 year and a major MR (MMR) rate of 85%.^20^

The objective of this phase II, open‐label, multicenter study was to investigate the optimal nilotinib treatment strategy after having started at an initial dose of nilotinib 300 mg BID in patients with newly diagnosed CP‐CML based on early achievement of MR^4.5^.

## 2 METHODS

### 2.1 Study design, treatments, and blinding

The phase II N‐Road study was an open‐label, multicenter clinical study which included individuals who were newly diagnosed with CML‐CP. Each patient received nilotinib 300 mg BID (600 mg/day) for a period of 24 months. In this study, increasing the nilotinib dose to 400 mg BID (800 mg/day) was allowed if patients satisfied the criteria for lack of optimal response at any time point. The criteria for lack of optimal response in this study were as follows: BCR‐ABL1^IS^ (*BCR‐ABL1/ABL1* ratio on the International Scale [IS]) >10% after 3 months; BCR‐ABL1^IS^ >1% or Philadelphia chromosome‐positive rate >0% via chromosome banding analysis (G‐band) or peripheral blood neutrophil‐fluorescence in situ hybridization (FISH) method after 6 months; BCR‐ABL1^IS^ > 0.1% after 12 months; two consecutive losses of MMR (BCR‐ABL1^IS^ > 0.1%); BCR‐ABL1^IS^ > 0.0032% after 18 months; or two consecutive losses of MR^4.5^ (BCR‐ABL1^IS^ > 0.0032%). Subjects were observed closely to detect hematotoxicity (decreased neutrophil, platelet count, and anemia), other toxicities, and QT prolongation. The dose of nilotinib could be reduced or discontinued, depending on the toxicity severity, according to dose adjustment criteria. The following four nilotinib dose reduction levels were set: Level 4, 400 mg BID (800 mg/day); Level 3, 300 mg BID (600 mg/day); Level 2, 400 mg QD (400 mg/day); and Level 1, 200 mg QD (200 mg/day).

This research was approved by the institutional review boards at Jikei University Kashiwa Hospital and other participating institutions and was conducted in accordance with the ethical guidelines for medical and health research involving human subjects. This study also adhered to the ethical principles of the Declaration of Helsinki. All patients provided written informed consent for participation prior to initiation of any study procedures. This trial was registered in the University Hospital Medical Information Network Clinical Trials Registry (UMIN000008565).

### 2.2 Patients

Major inclusion criteria were as follows: patients aged ≥16 years, diagnosed with CML (Philadelphia chromosome‐positive, confirmed by chromosome analysis, or having *BCR‐ABL* mRNA confirmed by reverse transcription‐polymerase chain reaction [RT‐PCR]) within 6 months of study registration; patients who did not meet any AP or BP criteria; and had Eastern Cooperative Oncology Group performance status of 0 to 2.

Key criteria resulting in exclusion from the study were as follows: patients undergoing TKI treatment other than imatinib; patients undergoing imatinib treatment for ≥2 weeks; patients undergoing interferon‐α treatment; patients receiving oral cancer drugs (eg, hydroxyurea) for ≥3 months; patients receiving other study drugs for CML; patients with *BCR‐ABL* point mutations (T315I); patients with history of hematopoietic stem cell transplantation; patients with uncontrollable cardiovascular conditions (for example, individuals with hypertension, congestive heart failure, or arrhythmia); patients with acute/chronic pancreatitis; patients with a digestive disorder; patients with a congenital/acquired bleeding disorder; patients with uncontrollable diabetes or hyperlipidemia; and patients with any other uncontrollable diseases.

### 2.3 Efficacy outcomes

The primary endpoint, as specified in the study analysis plan, was MR^4.5^ rate by 24 months. Secondary endpoints were as follows: cumulative incidence of MR^4.5^, MR^4^, and MMR at 12 months and at 24 months; MR^4.5^ rate by 12 and 18 months; rate of MR^4^ and MMR by 12, 18, and 24 months; major CCyR rate by 6 and 12 months; and survival (specifically progression‐free survival [PFS], overall survival [OS], and event‐free survival [EFS]).

For PFS, progression was defined in this study as disease exacerbation to AP/BP and was measured from the date of registration to the date of exacerbation or death. For EFS, events were defined as AP/BP progression or loss of response (either complete hematologic response, CCyR, or partial cytogenetic response); EFS was measured from the date of registration to the date of the event or death.

### 2.4 Safety

Adverse events (AEs) were collected up to the date of study treatment completion (or 30 days after discontinuation for the patients who discontinued). The reported AEs were classified and graded according to Common Terminology Criteria for Adverse Events v4.0, Japan Clinical Oncology Group.

### 2.5 Assessments

Drug exposure to nilotinib was measured in terms of treatment duration, total dose received, and dose intensity in all cases, as well as by achievement or nonachievement of optimal response. Testing of *BCR‐ABL1* gene levels was conducted; measurements were obtained using IS quantitative RT‐PCR performed in a central laboratory using a Molecular MD One‐Step qRT‐PCR BCR‐ABL kit (BML Inc).

Following the methodology used in a previously published nilotinib study,^21^ and using standardized definitions,^20^ MR^4^ was defined as detectable ≤0.01% BCR‐ABL1^IS^ or undetectable BCR‐ABL1 in samples with ≥10 000 ABL1 transcripts. In the case of undetectable BCR‐ABL1, samples with a mean of <10 000 ABL1 transcripts, or with a total of <10 000 ABL1 transcripts, were considered not evaluable for MR^4^. MR^4.5^ was defined as detectable ≤0.0032% BCR‐ABL1^IS^ or undetectable BCR‐ABL1 in samples with ≥32 000 ABL1 transcripts.^20^, ^21^ In the case of undetectable BCR‐ABL1, samples with a mean of <32 000 ABL1 transcripts, or with a total of <32 000 ABL1 transcripts, were considered unevaluable for MR^4.5^. No major MR was defined as a BCR‐ABL1^IS^ >0.1%. MMR was defined as a BCR‐ABL1^IS^ ≤0.1%. Loss of MMR was defined in this study as an increase in BCR‐ABL1^IS^ above >0.1%; this was reconfirmed after Week 4 by molecular genetic testing. Loss of MR^4.5^ was defined as a BCR‐ABL1^IS^ >0.0032% and this was reconfirmed after Week 4 by molecular genetic testing. Confirmation of the Philadelphia chromosome presence was made via the G‐band or the FISH method. MR^4.5^ duration was defined as the longest duration without MR^4.5^ loss (two successive losses, BCR‐ABL1^IS^ >0.0032%).

### 2.6 Statistical methods

Descriptive statistics were used for baseline demographic and clinical characteristics, with number (%) for categorical variables and median (range) for continuous variables. Kaplan‐Meier analysis was used to assess the cumulative incidence of MR^4.5^, MR^4^, MMR, PFS, OS, and EFS over 12‐ and 24‐month periods. The proportion of patients with CMR (considered the best response) during the follow‐up period (24 months after the start of nilotinib treatment) was analyzed. MR rates were analyzed by the best response at each time point. Accordingly, point estimation for the proportions and two‐sided confidence intervals (CI) and 95% confidence coefficients were calculated. Using Kaplan‐Meier methodology, the cumulative survival functions (ie, OS/PFS/EFS) were each determined and shown graphically. The estimated cumulative survival rate at 6, 12, 18, and 24 months and the two‐sided CI with 95% confidence coefficient were also calculated. The cumulative survival function was likewise determined using the Kaplan‐Meier method and depicted graphically. The time to achieve each specified event was summarized using descriptive statistics. SAS software version 9.4 (SAS Institute) was used for statistical analysis.

## 3 RESULTS

### 3.1 Patient disposition and baseline characteristics

**FIGURE 1:**
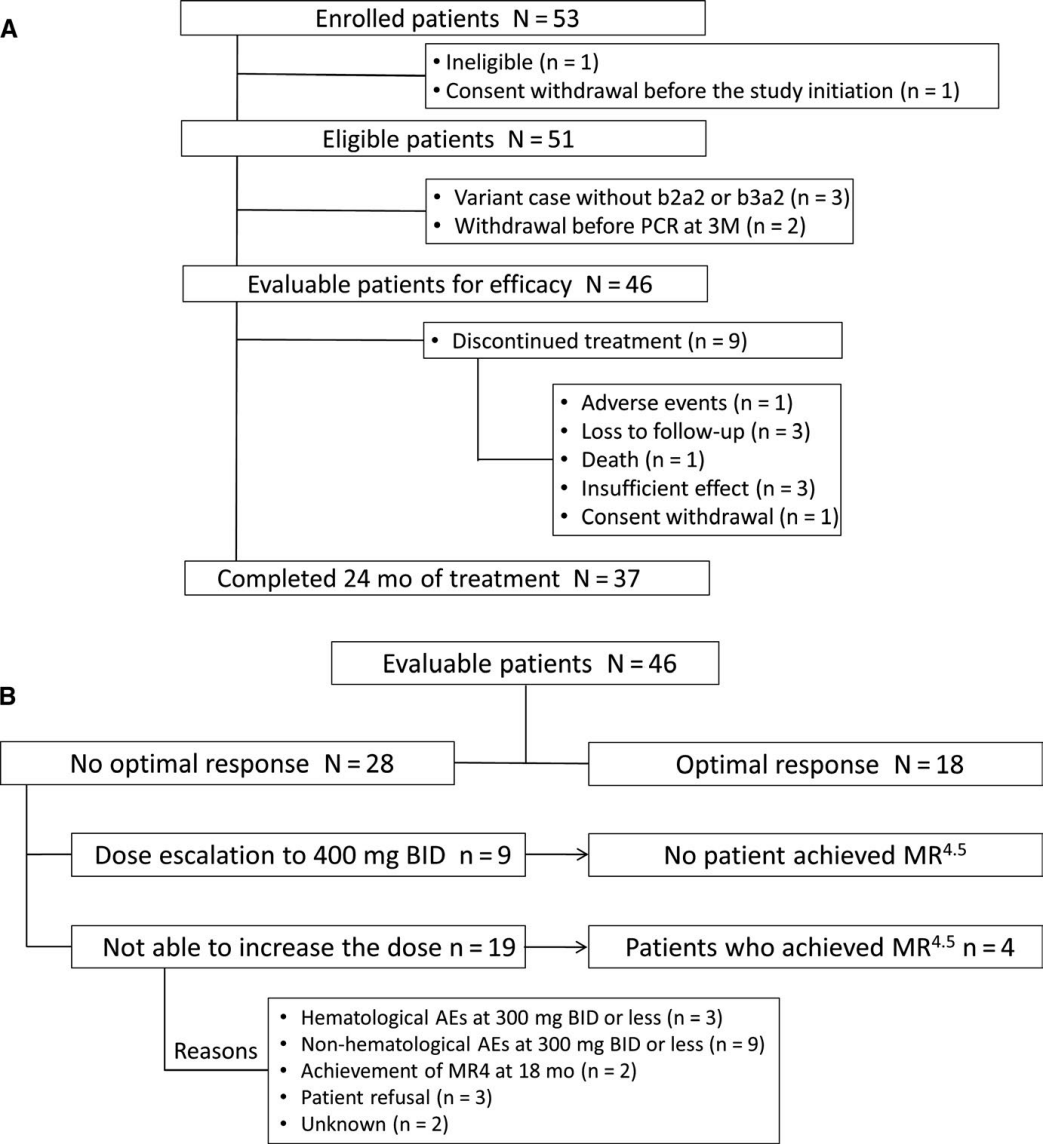
A, Patient disposition, and B, dose adjustment outcome. AE, adverse event; BID, twice daily; M, month; MR^4^, molecular response 4 log reduction; MR^4.5^, molecular response 4.5 log reduction; PCR, partial complete response

The study was conducted between August 2012 and July 2015 in 14 centers. Figure 1A shows the disposition of patients. A total of 53 patients were enrolled in this study. Of the 51 eligible patients, three patients with variant cases and two patients who withdrew from the study before PCR at 3 months were not included in the efficacy analysis; thus, 46 patients were evaluable for efficacy. Of these, 37 completed the 24‐month treatment, and nine discontinued treatment. The main reasons for treatment discontinuation were loss to follow‐up (n = 3) and insufficient effect (n = 3).

The main characteristics and clinical features of the patients included in the analysis sets for efficacy and safety are shown in Table 1. In the safety analysis set, patients had a median (range) age of 50 (24‐86) years and 35.3% were females. Patients in the safety analysis set had a predominantly low (41% and 43%) or intermediate risk (41% and 47%) according to the Sokal and Hasford risk scores, respectively. Most patients (76%) in the safety analysis set had low risk according to the European Treatment and Outcome Study risk score. As patients in this study were newly diagnosed with CML, per the inclusion criteria, the median disease duration was short (0.16 [0‐2.5] months; safety analysis set). Complications such as diabetes mellitus and hypertension were present in 11% and 20% of patients (safety analysis set).

**TABLE 1:** Demographic and clinical characteristics of patients at baseline

	Safety set (n = 51)	Efficacy set (n = 46)
Age, y	50 (24‐86)	48.5 (24‐86)
Sex, female	18 (35.3%)	16 (34.8%)
Sokal risk score		
Low	21 (41%)	20 (43%)
Intermediate	21 (41%)	18 (39%)
High	6 (12%)	6 (13%)
Unknown	3 (6%)	2 (4%)
Hasford risk score		
Low	22 (43%)	19 (41%)
Intermediate	24 (47%)	23 (50%)
High	1 (2%)	1 (2%)
Unknown	4 (8%)	3 (7%)
EUTOS risk score		
Low	39 (76%)	35 (76%)
High	8 (16%)	8 (17%)
Unknown	4 (8%)	3 (7%)
Prior treatment with HU	15 (29.4%)	14 (30.4%)
CML duration, months	0.16 (0‐2.5)	0.13 (0‐2.5)
Complications		
Diabetes mellitus	5 (11%)	5 (11%)
Hypertension	10 (20%)	8 (17%)
Previous history		
Peripheral arterial occlusive disease	0	0
Cerebrovascular disease	1 (2%)	1 (2%)
Ischemic heart disease	0	0
Smoker	11 (22%)	11 (24%)
Ex‐smoker	7 (14%)	7 (15%)
Non‐smoker	32 (63%)	27 (59%)
Unknown	1 (2%)	1 (2%)

Note

Data are presented as n (%) or median (range).

Abbreviations: CML, chronic myelogenous leukemia; EUTOS, European Treatment and Outcome Study; HU, hydroxyurea.

### 3.2 Treatment exposure

Of the 46 evaluable patients, 18 achieved an optimal response and 28 did not (Figure 1B). Of the latter, nine patients underwent dose escalation to 400 mg BID, but none achieved MR^4.5^. Of these nine patients, three required dose reduction/interruption from 300 mg BID before the dose could be increased. In two of these three cases, the dose could be increased immediately at the time no optimal response was established, but a dose reduction/interruption was required before the determination of no optimal response. Response in the one remaining patient was judged as no optimal response at 3 months, but the dose could not be increased due to an AE, and further reduction/interruption was required. At 12 months, in that one patient, the dose was finally increased from 300 mg BID to 400 mg BID because of no optimal response. Moreover, of these nine patients, no case required further dose reduction or interruption after the dose was increased.

The dose could not be escalated in the remaining 19 patients—12 (63%) because of AEs and 7 (37%) because of non‐AE related reasons (Figure 1B). However, four of these patients achieved MR^4.5^. The main reasons for not undergoing dose escalation were nonhematological AEs at 300 mg BID or less (n = 9), hematological AEs at 300 mg BID or less (n = 3), and patient refusal (n = 3).

Overall, the nilotinib treatment had a median (range) duration of 729 (176‐798) days, the median total dose was 436.5 (36.4‐508.2) g, and the median dose intensity was 600 (207‐736) mg/day. Among patients who had an optimal response, five (27.8%) required a dose reduction and three (16.7%) required treatment interruption. Among patients without optimal responses, 11 (39.3%) required a dose reduction and 11 (39.3%) required treatment interruption.

### 3.3 Primary and secondary endpoints

**FIGURE 2:**
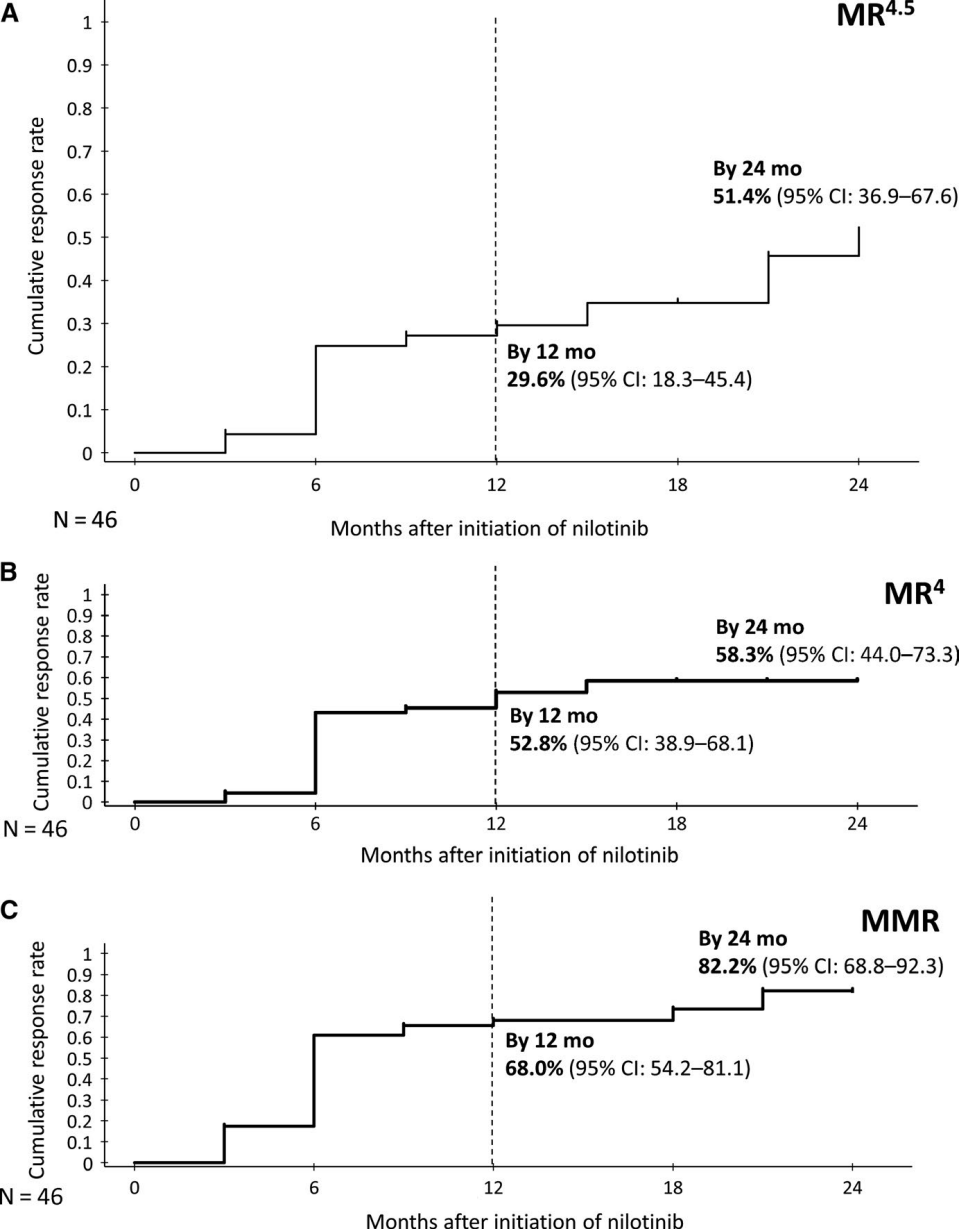
A, Cumulative incidence of MR^4.5^, B, Cumulative incidence of MR^4^, and C, Cumulative incidence of MMR. CI, confidence interval; MR^4^, molecular response 4 log reduction; MR^4.5^, molecular response 4.5 log reduction; MMR, major molecular response

The MR^4.5^ rate by 24 months (the primary endpoint) was 45.7% (95% CI: 30.9‐61.0). At 12 and 24 months, the cumulative incidence of MR^4.5^ was 29.6% (95% CI: 18.3‐45.4) and 51.4% (36.9‐67.6), respectively (Figure 2A). At 12 and 24 months, the cumulative incidence of MR^4^ was 52.8% (38.9‐68.1) and 58.3% (44.0‐73.3), respectively (Figure 2B). At 12 and 24 months, the cumulative incidence of MMR was 68.0% (54.2‐81.1) and 82.2% (68.8‐92.3), respectively (Figure 2C). By 12 and 18 months, 28.3% (95% CI: 16.0‐43.5) and 32.6% (19.5‐48.0) of patients achieved MR^4.5^, respectively. By 12, 18, and 24 months, 50.0% (95% CI: 34.9‐65.1), 54.3% (39.0‐69.1), and 54.3% (39.0%‐69.1%) of patients achieved MR^4^ and 65.2% (49.8‐78.6), 69.6% (54.2‐82.3), and 76.1% (61.2‐87.4) of patients achieved MMR, respectively. By 6 and 12 months, 87.0% (95% CI: 73.7‐95.1) and 89.1% (76.4‐96.4) of patients achieved CCyR, respectively.

**FIGURE 3:**
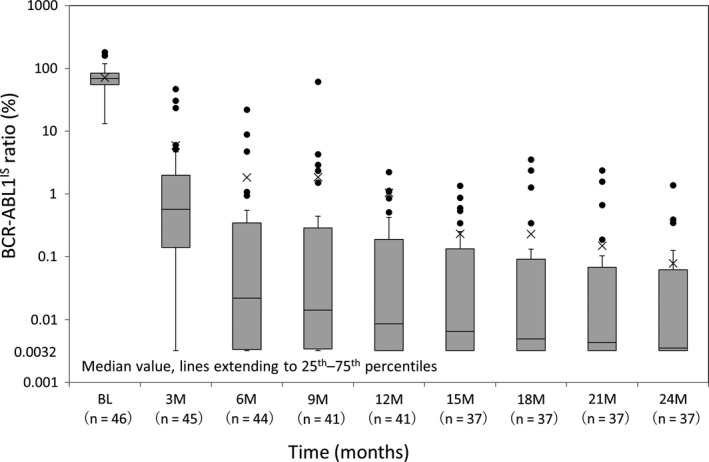
BCR‐ABL1^IS^ rate (%) over time. BL, baseline; IS, international scale; M, month

Figure 3 shows the transition of BCR‐ABL1^IS^ at each time point. The median BCR‐ABL1^IS^ decreased from a median of 69.4% at baseline to <1% until the end of the study (0.574 at 3 months, 0.022 at 6 months, 0.014 at 9 months, 0.009 at 12 months, 0.007 at 15 months, 0.005 at 18 months, 0.004 at 20 months, and 0.004 at 24 months)

### 3.4 Safety

Table 2 summarizes the AEs of any grade and Grade 3/4 reported during the study.

**TABLE 2:** Summary of adverse events (AEs) of any grade and grade 3/4

n = 51	Any grade	Grade 3/4
	n (%)	n (%)
Most frequent nonhematologic AEs (at least 10%)		
Rash	24 (47.1)	0 (0)
Headache	21 (41.2)	1 (2.0)
Nausea	12 (23.5)	0 (0)
Malaise	11 (21.6)	0 (0)
Fatigue	9 (17.6)	0 (0)
Myalgia	7 (13.7)	1 (2.0)
Upper respiratory tract infection	6 (11.8)	0 (0)
QTc prolongation	6 (11.8)	0 (0)
Other nonhematologic AEs of interest		
Fluid retention	7 (13.7)	2 (3.9)
Peripheral edema	4 (7.8)	0 (0)
Pericardial effusion	2 (3.9)	2 (3.9)
Pleural effusion	1 (2.0)	0 (0)
Vomiting	5 (9.8)	0 (0)
Arthralgia	5 (9.8)	1 (1.9)
Alopecia	5 (9.8)	0 (0)
Myocardial infarction	2 (3.9)	2 (3.9)
Death NOS	1 (2.0)	1 (2.0)
Hematologic abnormality (at least 5%)		
Anemia	40 (78.4)	0 (0)
Lymphopenia	33 (64.7)	0 (0)
Neutropenia	28 (54.9)	3 (5.9)
Thrombocytopenia	21 (41.2)	0 (0)
Leukopenia	14 (27.5)	0 (0)
Lymphocytosis	7 (13.7)	0 (0)
Biochemical abnormalities (at least 10%)		
Hyperglycemia	38 (74.5)	2(3.9)
Increased total bilirubin	32 (62.7)	1 (2.0)
Increased alanine aminotransferase	32 (62.7)	4 (7.8)
Hypophosphatemia	29 (56.9)	6 (11.8)
Hypertriglyceridemia	26 (51.0)	1 (2.0)
Increased gamma‐glutamyl transferase	25 (49.0)	3 (5.9)
Increased aspartate aminotransferase	23 (45.1)	1 (2.0)
Hypercholesterolemia	21 (41.2)	0 (0)
Decreased calcium	20 (39.2)	0 (0)
Increased lipase	15 (29.4)	5 (9.8)
Hypoalbuminemia	11 (21.6)	0 (0)
Increased alkaline phosphatase	10 (19.6)	0 (0)
Decreased potassium	10 (19.6)	0 (0)
Increased amylase	7 (13.7)	0 (0)
Decreased sodium	7 (13.7)	0 (0)
Increased potassium	7 (13.7)	0 (0)

Abbreviation: NOS, not otherwise specified.

The most common nonhematologic AEs reported with an incidence of ≥10% were rash, headache, nausea, malaise, fatigue, myalgia, upper respiratory tract infection, and QTc prolongation. Of these, one case of Grade 3/4 headache and one of myalgia were reported.

The most common hematologic AEs with an incidence of ≥5% were anemia, lymphopenia, neutropenia, thrombocytopenia, leukopenia, and lymphocytosis. Of these AEs, three cases of Grade 3/4 neutropenia were reported. Other Grade 3/4 AEs of interest were pericardial effusion and myocardial infarction, reported in two patients each, and death in a single patient. The patient who died seemingly had no health issues while under treatment but died suddenly. As no autopsy was performed, the cause of death was not confirmed. Other AEs were not unexpected.

## 4 DISCUSSION

The N‐Road study aimed to determine the optimal treatment strategy for nilotinib use in patients with newly diagnosed CML‐CP. The MR^4.5^ rate by 24 months was 45.7% in the present study, which is higher than the MR^4.5^ achieved in the 24‐month follow‐up of the ENESTnd trial (namely, 25% with a nilotinib dose of 300 mg BID).^19^, ^22^ MMR achievement was reported for 71% of nilotinib 300 BID‐treated patients in a 24‐month follow‐up of the ENESTnd trial,^19^, ^22^ which is comparable to the 76.1% rate of MMR achievement by 24 months in the present study. The cumulative incidences of MR^4.5^, MR^4^, and MMR with 300 mg BID nilotinib at 24 months were higher in this study than that of the ENESTnd trial (51.4% vs 25%, 58.3% vs 39%, and 82.2% vs 71%, respectively).^19^ In the ENEST1st trial, which assessed nilotinib 300 mg BID for patients with newly diagnosed CML‐CP,^21^ the cumulative incidences of MR^4.5^, MR^4^, and MMR by 24 months were 38.6%, 55.2%, and 80.4%, respectively. The response rate with nilotinib was similar to that in this study, but the cumulative incidence of MR^4.5^ by 24 months was higher in the current study.

Compared with the 5‐year follow‐up of the ENESTnd trial,^19^ we found that the incidence of MR^4.5^ in individuals who had a high Sokal score was lower than that of individuals who had a low or intermediate Sokal score (28% ENESTnd,^11^ 18.1% ENEST1st,^21^ and 13%, N‐Road). The treatment efficacy in the N‐Road study was different from other studies possibly because there were fewer patients with a high Sokal score in this study. Additionally, the clinical experience gained since the ENESTnd trial^11^ was conducted has led to improved management of patients treated with nilotinib. This difference may have influenced the results in this study and the ENEST1st trial.^21^ However, it is not possible to directly compare these results as the conditions (ie, selection criteria, patients’ background, number of patients, and dose reduction criteria) differed across studies. Importantly, a dose increase of >300 mg BID was allowed in the N‐Road study while this was not allowed in the ENESTnd and ENEST1st trials.^11^, ^21^

Our study had similar dose increase criteria (>300 mg BID) to two other nilotinib studies of patients with newly diagnosed CML‐CP: the ENESTxtnd^23^ and GIMEMA^24^, ^25^ trials. In the ENESTxtnd trial,^23^ 20.9% of patients had their nilotinib dose increased when they showed a suboptimal response to treatment and by 24 months, 63.6% of those patients were shown to have achieved MMR. A total of 17.6% of patients decreased doses because of AEs. The cumulative incidence of MMR was 81% by 24 months.^23^ Conversely, in the GIMEMA trial,^25^ 77% of patients continued nilotinib up to the last observation. Most patients (68%) received nilotinib 300 mg BID and 3% received 400 mg BID. The cumulative incidences of MR^4^ and MR^4.5^ by 2 years were 51% and 24%, respectively.^25^

Compared with those two trials, the N‐Road study showed a deeper MR. The N‐Road study aimed to achieve early response; thus, the dose increase criteria for no optimal response at 3, 6, and 12 months recommended by ELN 2013 was applied in this study.^26^ The dose increase criteria applied in N‐Road were, therefore, even stricter than those of ENESTxtnd^23^ and GIMEMA trials.^24^, ^25^ Consequently, the cumulative rate of MR^4.5^ by 24 months was high in our study. A total of 28 patients met the dose increase criteria, but only nine patients tolerated an increased dose. Of these nine patients, none achieved MR^4.5^. The remaining 19 patients could not undergo dose escalation; the main reason was AEs (12 [63%] patients), followed by non‐AE related reasons (7 [37%] patients). Although these 19 patients could not undergo dose escalation, MR^4.5^ was achieved by four (21%) of them. Thus, applying stricter criteria for dose increase compared with other trials may not have contributed to a high MR^4.5^ rate by 24 months of treatment in this study.

Regarding patient background characteristics in the ENESTxtnd trial,^23^ GIMEMA trial,^25^ and the present N‐Road study, the median age (range) was comparable. The rates of high Sokal score were no data,^23^ 21%,^25^ and 13%, respectively. A higher MR^4.5^ accumulative rate in this study may have been a result of the inclusion of fewer individuals who had a high Sokal score within our sample. The dose reduction criteria of this study were different from those applied in the ENESTxtnd trial.^23^ However, in both studies, the dose discontinuation criteria were the same, and the dose intensity in this study was similar to the other studies.^23^, ^25^

The curve for the cumulative rate of MMR was steeper at 6 months in this study compared with the ENESTxtnd trial.^23^ Patients in this study achieved a deep MR earlier than in some prior studies.^23^ The ENESTxtnd trial results imply many patients with poor early response could achieve MMR by increasing the nilotinib dose to 400 mg BID. Of those patients who did not achieve MMR or had a loss of MMR at 12 months of treatment, it was reported that 80% achieved MMR at 24 months when the dose was increased.^23^ Of patients with BCR‐ABL1^IS^ >10% at 3 months of treatment, only one‐third of patients achieved MMR.^24^ BCR‐ABL1^IS^ ≤10% at 3 months of treatment is an important milestone to achieve an optimal response.^27^, ^28^, ^29^ In the ENEST1st trial,^21^ none of the patients with BCR‐ABL1^IS^ >10% at 3 months of nilotinib treatment achieved MR^4.5^ at 2 years; 14.5% and 45.8% of patients with BCR‐ABL1^IS^ >1% to ≤10% and BCR‐ABL1^IS^ > 0.0032% to ≤1% at 3 months of nilotinib treatment achieved MR^4.5^ at 2 years, respectively. BCR‐ABL1^IS^ >0.0032% to ≤1% at 3 months of treatment seems to be a significant factor for the achievement of early MR^4.5^. The rates of BCR‐ABL1^IS^ >0.0032% to ≤1% at 3 months in the ENEST1st^21^ and N‐Road studies were 78.5% and 58.1%, respectively; those with BCR‐ABL1^IS^ >1% to ≤10% were 18.5% and 30.2%, respectively; and those with BCR‐ABL1^IS^ >10% were 2.9% and 11.6%, respectively. The rate of MR^4.5^ at 2 years in the N‐Road study was higher than that in the ENEST1st trial although the rate of BCR‐ABL1^IS^ >10% was higher and that of BCR‐ABL1^IS^ >0.0032% to ≤1% was lower in the N‐Road study; however, the reason for this difference in results is unknown.

The safety of nilotinib in this study was similar to that reported in previous studies.^19^, ^21^, ^25^ In this study, two patients (3.9%) presented cardiovascular events, both grade 3 myocardial infarction. We did not record any cases of ischemic cerebrovascular disease. In the ENEST1st trial,^21^ during 2 years of treatment, 3.4% of patients had ischemic heart disease, and 0.8% had ischemic cerebrovascular disease. In the GIMEMA trial,^25^ 4.6% of patients presented an arterial thromboembolic event. In this study, patients who had myocardial infarction or unstable angina pectoris within the prior 12 months immediately before study registration were excluded. After two patients presented myocardial infarction in this study, patients with congestive heart failure, uncontrollable diabetes, or hyperlipidemia were excluded from this study. After this change, no obstructive cardiovascular events were observed. Thus, we consider that cardiovascular events may be prevented by controlling risk factors such as diabetes, hyperlipidemia, blood pressure, and smoking,^30^ making it possible to continue nilotinib treatment.

This study had some limitations, such as those related to the open‐label nonrandomized study design. Additionally, the sample size was limited, and the study lacked a comparator; thus, the generalizability of the results may be limited. Finally, this study aimed to assess the optimal treatment strategy with nilotinib; however, dose escalations were not possible in several patients, which precluded dose‐escalation analyses.

Notably, there were many cases in which an early dose increase was difficult because of AEs. In other cases, even if the dose could be increased, MR^4.5^ could not be reached in the 24‐month period. While the results of this study do not support an early dose increase of nilotinib for individuals who do not manage to achieve an optimal response, most patients in our study received nilotinib 300 mg BID. By optimizing the nilotinib dose in cases requiring dose increases or decreases, and by managing AEs, treatment continuation was successful at this dose. The results of the N‐Road study suggest the importance of continuing nilotinib treatment at a dose of 300 mg BID, as it may lead to the achievement of early deep MR in patients with newly diagnosed CML‐CP.

## DISCLOSURE STATEMENT

KN received a grant for this work from Novartis Pharma KK; and reports grants from Zenyaku Kogyo Company, Ltd., Asahi Kasei Pharma, and Taiho Pharmaceutical Co., Ltd., grants and personal fees from Chugai Pharmaceutical, Kyowa Hakko Kirin Co., Ltd., Nippon Shinyaku Co., Ltd., Mochida Pharmaceutical Co., Ltd., Ono Pharmaceutical Co., Ltd., Takeda Pharmaceutical Co., Ltd., and Sumitomo Dainippon Pharma Co., Ltd., and personal fees from Pfizer, Otsuka Pharmaceutical Co., Ltd., Janssen Pharmaceutical KK, Eisai Co., Ltd., and Celgene KK, outside the submitted work. HY reports grants from Celgene KK and Astellas Pharma Inc, outside the submitted work. AT reports personal fees from Sysmex, Otsuka Pharmaceutical Co., Ltd., Taiho Pharmaceutical Co., Ltd., Bristol‐Myers Squib, and Takeda Pharmaceutical Co., Ltd., and grants and personal fees from Daiichi Sankyo, Pfizer, and Chugai Pharmaceutical, outside the submitted work. KS, ST, JH, TI, AS, TS, SH, KF, SS, TY, and HW have nothing to disclose.

## AUTHOR CONTRIBUTIONS

KN, AT, and HW designed the study. KN, KS, AT, and HW analyzed and interpreted the data. KN developed the manuscript and KN, AT, and HW revised the manuscript extensively. All authors participated in data collection and approved the final manuscript for submission.

## Data Availability

The data that support the findings of this study are available on request from the corresponding author. The data are not publicly available due to privacy or ethical restrictions.
